# Magnesium Depletion Score Predicts Diabetic Retinopathy Risk among Diabetes: Findings from NHANES 2005–2018

**DOI:** 10.1007/s12011-022-03384-3

**Published:** 2022-08-22

**Authors:** Yuan Chen, Xiaoli Xiang, Yangyang Wu, Shaojie Han, Zhengru Huang, Miaoqin Wu

**Affiliations:** 1 Soochow University, 215031 Suzhou, China; 2 Department of Ophthalmology, Changshu No. 2 People’s Hospital, Changshu, 215500 China; 3 Department of Ophthalmology, Zhejiang Provincial People’s Hospital, Hangzhou, 310014 China; 4 Bengbu Medical College, Bengbu, 233030 China; 5 Gushu College, Nanjing Medical University, Nanjing, 211166 China

**Keywords:** Magnesium, Magnesium depletion score (MDS), Diabetes mellitus (DM), Diabetic retinopathy (DR)

## Abstract

Magnesium is essential for material and energy metabolism. The magnesium depletion score (MDS) is recognized as a more valuable and reliable predictor of body magnesium status than any other clinical used markers such as serum and urine magnesium. However, research on the relationship between MDS and diabetic retinopathy (DR) is limited. As a result, the current study sought to assess this issue in diabetic samples from a large population-based database in the United States. Data were obtained from the National Health and Nutrition Examination Survey (NHANES) 2005–2018. MDS was calculated, and multivariate logistic regression analysis was applied to evaluate the presence of association between variables and DR risk. A total of 4308 participants was comprised in this study. Samples with DR consumed less magnesium (259.1 ± 113.6 vs 269.8 ± 113.2 mg, *P* < 0.001), and their MDS levels differed significantly from non-DR participants (*P* < 0.001). Increased dietary magnesium was linked to a lower incidence of DR (all *P* for trend < 0.05), and patients with a high level of MDS were more prone to DR (*P* = 0.001). Furthermore, subgroup analysis revealed that high (Q3) amount magnesium supplements was associated with lower DR risk when MDS was none to low or middle level (both *P* = 0.02). Our results indicated that MDS levels are associated with DR risk and that magnesium supplementation is benefit to DR prevention.

## Introduction

Diabetic retinopathy (DR) is a major cause of blindness in people of working age and is one of the principal visual complications associated with long-term diabetes mellitus (DM) [1]. Although various strategies are applied for preventing and treating DR, vision preservation remains a challenge [2]. To date, multiple independent risk factors in DR progression have been discovered [3, 4], which might serve as targets to regulate and prevent DR progression; nonetheless, the discussion and discovery on DR etiology continues. More recently, several researches have shown that minerals, notably magnesium, may have a role in DR prevention.

Magnesium, one of the most essential minerals in the body, serves as a cofactor for over 350 enzymes and is involved in a variety of physiological and pathological processes [5, 6]. According to the Estimated Average Requirement, more than half of persons in the United States do not meet the magnesium consumption threshold [7]. Although serum magnesium is often used in clinical applications to assess magnesium shortage [8, 9], it may not accurately represent the entire magnesium state of the body as the kidneys ultrafiltrate and resorb over 80% of the magnesium in plasma, which plays an important role in maintaining magnesium homeostasis [10, 11]. The magnesium depletion score (MDS) index was recently proposed as a method of measuring magnesium shortage that took into consideration the pathophysiological factors influencing the kidneys’ reabsorption capability and was proven to be more sensitive and reliable than other clinical predictors of magnesium deficiency, such as serum magnesium and urine magnesium [11]. As a result, MDS was used in the current study for analysis in order to get a more solid connection between magnesium and DR risk.

The National Health and Nutrition Examination Survey (NHANES) is a national program in the United States aimed to measure adult and child nutrition and health status. The program examines or interviews around 5000 persons each year. Data from this survey can be utilized in medical studies for health promotion and disease prevention. In this study, we obtained and analyzed more than 10 years of NHANES data to establish the link between magnesium and DR.

## Methods

### Data Source

The current study collected data from the NHANES throughout seven cycles (2005–2018), and each participant provided informed consent. For further analysis, demographic, examination, laboratory, and questionnaire data were employed.

### Evaluation and Assessment of Diabetes

According to the Standards of Medical Care in Diabetes, such people was defined as having DM: (i) fasting plasma glucose (FPG) ≥ 126 mg/dL (7.0 mmol/L), (ii) 2-h plasma glucose (2-h PG) ≥ 200 mg/dL (11.1 mmol/L) during the oral glucose tolerance test (OGTT), and (iii) HbA1c ≥ 6.5% (48 mmol/mol). Moreover, those who replied “Yes” to the question “Did the doctor tell you that you have diabetes?” or who have used an antidiabetic drug in the past would also be recognized. DR was classified as those who answered “Yes” to the question “Diabetes affected eyes/had retinopathy.” The duration of diabetes was divided into two categories: 0–10 years and > 10 years. Glycemic control was classified as either good (HbA1c < 7%) or poor (HbA1c ≥ 7%).

### Assessment of MDS and Magnesium Intake

MDS was calculated to evaluate the total body magnesium status as described elsewhere [11]. In a nutshell, four factors were incorporated and aggregated: (1) diuretic application (one point for current application), (2) proton pump inhibitor application (one point for current application), (3) renal function (one point for 60 mL/min/1.73 m^2^ ≤ estimated glomerular filtration rate [eGFR] < 90 mL/min/1.73 m^2^; two points for eGFR < 60 mL/min/1.73 m^2^), and (4) alcohol consumption (one point for heavy drinker). MDS were further divided into three categories in the current study for ease of analysis and application: “none to low” (MDS = 0 or 1), “middle” (MDS = 2), and “high” (MDS > 2).

The quantity and types of food and beverages consumed were calculated using NHANES data from two 24-h recall interviews. The first 24-h personal interview was conducted in the Mobile Examination Center (MEC), and the second was conducted by telephone around 3–10 days later, according to the survey procedure. The total dietary magnesium elements were estimated by averaging two 24-h dietary recalls, and the amount of magnesium was separated into three levels: Q1 (≤177.5 mg), Q2 (177.6–316.0 mg), and Q3 (≥316.1 mg) for data analysis.

### Definition and Assessment of the Covariates

The categories of covariates were performed through NHANES definition and available original data classification. Participants were separated into four age groups: < 40, 40–59, 60–79, and ≥ 80 years old. Marital status was profiled as married and others. Moreover, homeostasis model assessment was evaluated using the homeostasis model assessment-estimated insulin resistance (HOMA-IR). In addition, those who answered “Yes” to the question “Ever told by a physician with difficulty in sleeping?” or “Ever told by a physician with a sleep disorder?” were classified as having “Sleep disorder.” Smoking status was divided into three categories: never (individuals who smoked less than 100 cigarettes in their lifetime), former (individuals who smoked more than 100 cigarettes in their lifetime but no longer smoke), and now (individuals who smoked more than 100 cigarettes in their lifetime but no longer smoke). Furthermore, hypertension was defined as a blood pressure reading over 140/90 mmHg recorded three times. In addition, the body mass index (BMI) was separated into three categories: <25.00, 25.00–29.99, and ≥30.00 kg/m^2^. NHANES used the patient health questionnaire (PHQ-9) depression scale to assess the depressive condition of participants. According to previous research [12], the PHQ-9 score ≥ 10 had a specificity and sensitivity of 88% and 88%, respectively, for severe depression. As a result, if the PHQ-9 score ≥ 10, we classed depression symptoms as “clinically significant.”

### Statistical Analysis

The current investigation used R Studio software (version 4.1.2) in accordance with NHANES analytic criteria. The “compareGroups” and “survey” packages were applied in this work. The comparison for categorical variables between DR and non-DR was performed using chi-square test. HOMA index was calculated and compared using Student’s *t*-test. Logistic regression analysis was performed to examine the presence of association between magnesium consumption or MDS and DR. The crude model only included dietary magnesium or MDS, whereas the adjusted model included the crude model plus general clinical characteristics like sex, age, marital status, and risk factors for DM or DR like BMI, DM duration, HOMA-IR, glycemic control, hypertension, and adverse lifestyles like sleep disorders and depression status.* P* for trend values were evaluated by treating categorical variables as ordinal. *P* < 0.05 (two-sided) stood for statistical significance.

## Results

### Baseline Information of Participants

**Fig. 1:**
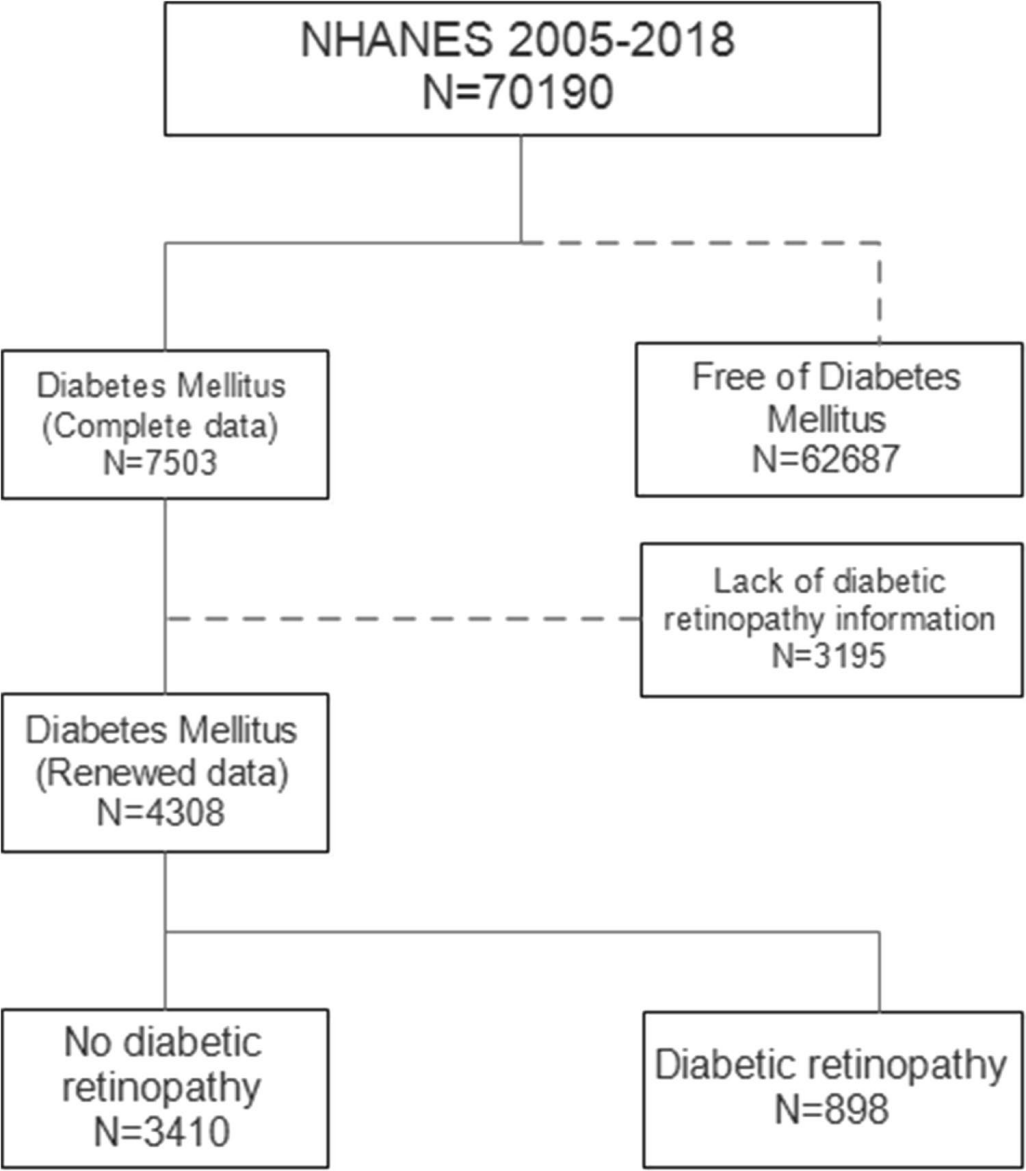
Flowchart showing the participants enrollment procedure

A total of 70,190 individuals were enrolled in this study, with 7503 participants meeting the DM criteria. We finally included 4308 samples after excluding those with insufficient DR information, shown as flowchart in Fig. 1. The estimated prevalence of DM was 10.7% (95% CI: 10.5–10.9%). Table 1 shows the demographic factors and their relationships with DR. Depression, diabetes duration, glycemic control, hypertension, marital status, HOMA-IR, and sleep problems all had significant differences in the distribution between two groups.

**Table 1:** Summary characteristics of participants by DR

	Non-DR	DR	*P* overall
	*N* = *3410*	*N* = *898*	
Age			0.034
< 40	257 (7.54%)	44 (4.90%)	
40–59	1041 (30.53%)	269 (29.96%)	
60–79	1788 (52.43%)	501 (55.79%)	
≥ 80	324 (9.50%)	84 (9.35%)	
Gender			0.173
Female	1673 (49.06%)	417 (46.44%)	
Male	1737 (50.94%)	481 (53.56%)	
Marital status			0.008
Married	1898 (56.40%)	459 (51.34%)	
Others	1467 (43.60%)	435 (48.66%)	
Race			0.115
Mexican American	606 (17.77%)	155 (17.26%)	
Non-Hispanic Black	921 (27.01%)	260 (28.95%)	
Non-Hispanic White	1248 (36.60%)	291 (32.41%)	
Other Hispanic	331 (9.71%)	99 (11.02%)	
Other race	304 (8.91%)	93 (10.36%)	
BMI			0.926
< 25.00	438 (13.09%)	117 (13.57%)	
25.00–29.99	942 (28.15%)	243 (28.19%)	
≥ 30.00	1966 (58.76%)	502 (58.24%)	
Diabetes duration, years			< 0.001
0 ~ 10	2086 (61.72%)	336 (37.58%)	
> 10	1294 (38.28%)	558 (62.42%)	
Glycemic control			< 0.001
Good	1752 (53.45%)	342 (39.91%)	
Poor	1526 (46.55%)	515 (60.09%)	
Hypertension			0.001
No	889 (26.07%)	172 (19.15%)	
Yes	2521 (73.93%)	726 (80.85%)	
HOMA-IR	8.24 (14.79)	10.82 (21.88)	0.026
Depression status			< 0.001
Not significant	2817 (87.73%)	694 (82.52%)	
Clinical significant	394 (12.27%)	147 (17.48%)	
Sleep disorder			0.012
No	1379 (51.34%)	327 (45.93%)	
Yes	1307 (48.66%)	385 (54.07%)	
Smoke			0.217
Never	1665 (49.44%)	447 (49.94%)	
Former	1171 (34.77%)	327 (36.54%)	
Now	532 (15.80%)	121 (13.52%)	

DR, diabetic retinopathy; BMI, body mass index; HOMA-IR, homeostatic model assessment of insulin resistance

**Fig. 2:**
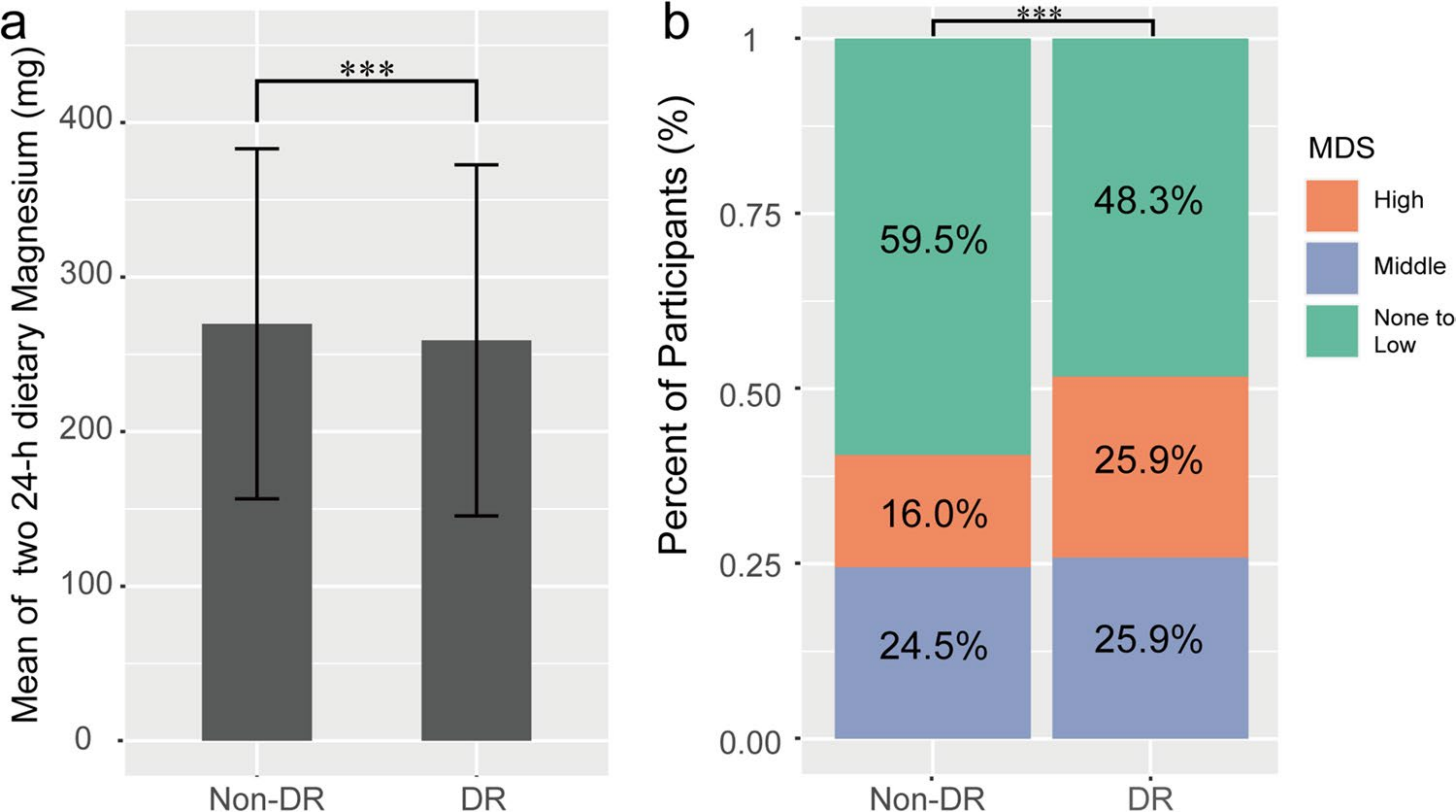
The amount of 24-h magnesium intake and the MDS distribution in DR and non-DR groups. **a** The average 24-h magnesium intake of DR and non-DR groups; **b** the MDS distribution of DR and non-DR groups. MDS, magnesium depletion score; DR, diabetic retinopathy; ***P < 0.001

The distribution of dietary magnesium or MDS in non-DR and DR groups is depicted in Fig. 2. It reveals that DR individuals consumed less magnesium than non-DR participants (259.1 ± 113.6 vs 269.8 ± 113.2 mg, *P* < 0.001) and the MDS distribution of DR was of significant difference from non-DR (*P* < 0.001).

### Sensitivity Analyses

**Fig. 3:**
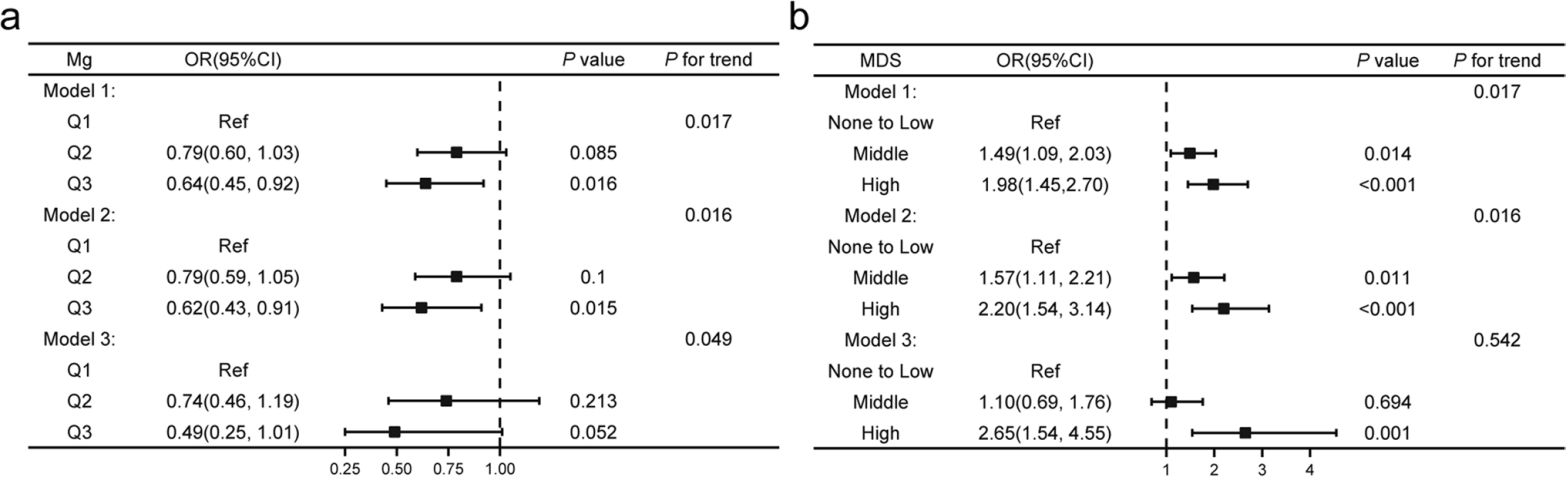
Sensitivity analysis of magnesium or magnesium depletion score (MDS) in different models. **a** Sensitivity analysis of magnesium intake; **b** sensitivity analysis of MDS. MDS, magnesium depletion score; OR, odds ratio; Ref = reference

A sensitivity test was used in our study to confirm the relevance of MDS or dietary magnesium with DR by controlling multiple variables. Model 1 was regarded as a crude model. Model 2 was adjusted based on model 1 plus sex, age, and marital status to exclude potential influence factors from the samples’ backgrounds. Furthermore, in model 3, variables linked with DR or DM, such as BMI, diabetes duration, hypertension, HOMA-IR, depression, and sleep disturbances, were further controlled. Figure 3a displays the relationship between dietary magnesium and the risk of DR. In all three models, the significance of *P* for trend suggested that increasing dietary magnesium was associated with a decreased DR risk (all *P* < 0.05). Furthermore, we discovered that in model 1 and model 2, high dosage (Q3) magnesium consumption was inversely associated with DR risk versus low dose (Q1). In addition, a borderline trend of Q3 was observed in model 3 although it was not statistically significant. The link between MDS and DR is depicted in Fig. 3b. *P* for trend was significant in models 1 and 2, but not in model 3. Moreover, in models 1 and 2, the results showed an elevated DR risk in middle and high MDS versus none to low MDS, but only the high level was significant and consistent in crude and all adjusted models, implying that higher MDS levels are connected to increased DR risk.

### Subgroup Analysis

Subgroup analysis was utilized to investigate the relationship between magnesium consumption and DR risk in patients with varied levels of magnesium deficiency in adjusted model. As indicated in Table 2, high dosage magnesium consumption (Q3) was associated with a reduced risk of DR when MDS was none to low or middle level. Furthermore, a notable trend of moderate magnesium intake (Q2) was identified when MDS equal to none to low, although it was not statistically significant. No correlation can be found in other subgroups. *P* for trend was significant when MDS was none to low or middle but not high. The foregoing findings imply that as the amount of magnesium insufficiency rises, more magnesium supplementation is required to minimize the risk of DR.

**Table 2:** Subgroup analysis of MDS and magnesium intake in relation to DR risk

MDS	Magnesium	OR (95% CI)	*P*	*P* for trend
None to low				0.025
	Q2 vs Q1	0.52 (0.26, 1.03)	0.060	
	Q3 vs Q1	0.34 (0.14, 0.84)	0.020	
Middle				0.021
	Q2 vs Q1	0.83 (0.36, 1.94)	0.660	
	Q3 vs Q1	0.22 (0.06, 0.78)	0.020	
High				0.823
	Q2 vs Q1	0.76 (0.33, 1.75)	0.512	
	Q3 vs Q1	0.97 (0.28, 3.43)	0.966	

## Discussion

This study evaluated the correlation of magnesium status or magnesium intake with DR based on MDS, a novel diagnostic for magnesium deficiency. Our results identified an adverse association between magnesium deficiency and DR risk and that adequate magnesium intake can benefit to DR prevention.

In this study, traditional risk variables for DR or DM were evaluated and further adjusted for logistic models. HBP [13], diabetes duration [14], glycemic control [14], sleep disorders [4], depression [15], and HOMA-IR score [16] were included as factors in our study, and the correlations were further verified in our analysis. Despite the fact that BMI has been suggested to be an independent risk factor for DM [17], our findings showed no link between BMI and DR, which is consistent with prior research [18].

Magnesium deficiency contributes to DM, which puts diabetic individuals at risk for DR. Low magnesium consumption has been linked to an increased risk of type 2 diabetic mellitus (T2DM) [19], while adequate magnesium supplementation has been linked to DM remission in obese people [20]. In target cells, intracellular magnesium concentrations were important in phosphorylating insulin receptors and other downstream signal kinases. Under the effect of insulin, magnesium deficiency causes tyrosine kinase activity to be disrupted, cell glucose transport to be altered, cell glucose consumption to be lowered, and post-receptor function to be compromised, all of which increase peripheral insulin resistance in T2DM [21] and could be a driver of retinopathy [16].

The research on the molecular mechanisms of magnesium on DR is limited, but various pathologies have been demonstrated. The ion’s physiological role included ATP generation and hydrolysis, as well as mediating the activity of the Na^+^/K^+^ and Na^+^/Ca^2+^ ATPases to maintain ionic homeostasis in the retina. Magnesium deficiency would contribute to increased vascular smooth Ca^2+^, endothelin-1, and thromboxane A2 and decreased prostacycline, which potentially result in vasoconstriction and retinal ischemia. Moreover, low magnesium levels would also enhance oxidative stress and elevate inducible nitric oxide synthase (iNOS), leading to the initiation and progression of DR [22].

Previous research on the association between retinopathy and magnesium has yielded varying results. According to Xing [8] and Oost [9], patients with low serum magnesium levels are more likely to experience DR. In contrast, Lima [23] and Xu [24] found no correlation between magnesium levels and DR. The results of the current study are consistent with the former. The disparities in results may be explained in part by the differences in serum magnesium measurement, heterogeneity of the study population, and different study designs. The use of MDS in this study may lead to a more solid conclusion as a more reliable predictor of magnesium shortage [11].

There are several limitations to our study that should be noted. Because of the cross-sectional form of NHANES, determining the causal influence can be difficult. In contrast, precise quantification of dietary nutrients intake and retinopathy status is difficult to recollect, and data collection via in-person interviews may be subject to misclassification bias.

In conclusion, our research indicates that magnesium deficiency predicts a higher risk of DR in diabetic individuals and that magnesium supplementation may reduce the risk of DR. Because this was a cross-sectional observational study, future large-scale cohort studies and biological tests are needed to corroborate these findings.

## Data Availability

The datasets generated during and/or analyzed during the current study are available at NHANES website, https://www.cdc.gov/nchs/nhanes/index.htm.
